# Acute Cerebral Infarction Secondary to Spontaneous Isolated Middle Cerebral Artery Dissection: A Case Report

**DOI:** 10.7759/cureus.99958

**Published:** 2025-12-23

**Authors:** Yoshiyuki Takada, Etsusho Matsunaga, Yuzo Saito, Yoshinobu Sekihara, Norihiro Ishii

**Affiliations:** 1 Department of Neurosurgery, New Tokyo Hospital, Matsudo, JPN

**Keywords:** acute ischemic stroke, headache, mechanical thrombectomy, neuroendovascular therapy, percutaneous transluminal angioplasty, spontaneous isolated middle cerebral artery dissection

## Abstract

Spontaneous isolated middle cerebral artery dissection (MCAD) is uncommon. We report a rare case of acute M1 occlusion resulting from spontaneous isolated MCAD. A 64-year-old man was admitted to our hospital with an abrupt onset of headache, left hemiparesis, and sensory disturbances. Magnetic resonance imaging revealed a cerebral infarction in the right hemisphere, and magnetic resonance angiography confirmed right M1 occlusion. The patient underwent mechanical thrombectomy and percutaneous transluminal angioplasty, achieving incomplete recanalization with residual stenosis. Retrospective analysis of angiography after the initial pass revealed findings suggestive of MCAD, indicating spontaneous isolated dissection. Reports of mechanical thrombectomy in patients with complete occlusion due to spontaneous isolated MCAD are limited, and the optimal treatment strategy remains unclear. Although diagnosing this rare condition is challenging, the presence of headache at onset may indicate MCAD. This case highlights the importance of considering spontaneous isolated MCAD as a possible cause of acute M1 occlusion and underscores the need for careful assessment of clinical history and imaging findings.

## Introduction

Intracranial artery dissection represents a crucial etiology of stroke. Nonetheless, isolated middle cerebral artery dissection (MCAD) is an uncommon occurrence [1-3], and its pathophysiology and optimal treatment strategy remain inadequately investigated. The latest European Stroke Organization guidelines reviewed only a single case of MCAD [4].

Given that MCAD rarely causes complete occlusion of M1 [3], diagnosing MCAD accurately and devising recanalization strategies for patients with complete M1 occlusion upon emergency admission pose challenges. Conversely, dissection typically manifests with headache; however, the frequency of headache in ischemic stroke overall is relatively low [5]. The presence of headache at onset could serve as a red flag for suspecting MCAD. This case report details an incidence of right-sided MCAD-triggered acute M1 occlusion with concomitant left-sided paralysis and headache, which necessitated mechanical thrombectomy (MT).

This article was previously presented as an oral presentation at the 50th Annual Meeting of the Japan Stroke Society (STROKE2025) on March 8, 2025.

## Case presentation

A 64-year-old man with a history of hypertension was admitted to our hospital with a sudden-onset headache and left hemiparesis. Upon arrival, he was alert but continued to experience a headache along with left incomplete hemiparesis, sensory disturbances, and left homonymous hemianopsia, with a National Institutes of Health Stroke Scale score of 10 [6].

**Figure 1:**
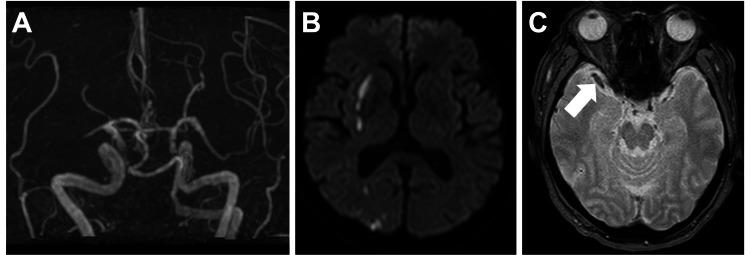
Imaging findings at admission (A) Magnetic resonance angiogram time-of-flight illustrating right MCA M1 occlusion and (B) diffusion-weighted image displaying acute cerebral infarction in the right MCA territory. (C) A susceptibility vessel sign-like finding with heterogeneous intensity was observed in the right M1 (white arrow). MCA: middle cerebral artery

An electrocardiogram taken at admission indicated a sinus rhythm. Magnetic resonance angiography (MRA) time-of-flight revealed an occlusion of the M1 segment in the right middle cerebral artery (MCA) (Figure 1A), whereas MRI revealed an acute cerebral infarction spanning from the right temporal lobe to the frontal and parietal lobes (Figure 1B). The MRI T2*-weighted image displayed a susceptibility vessel sign (SVS) in the right M1, but with a heterogeneous internal signal (Figure 1C).

**Figure 2:**
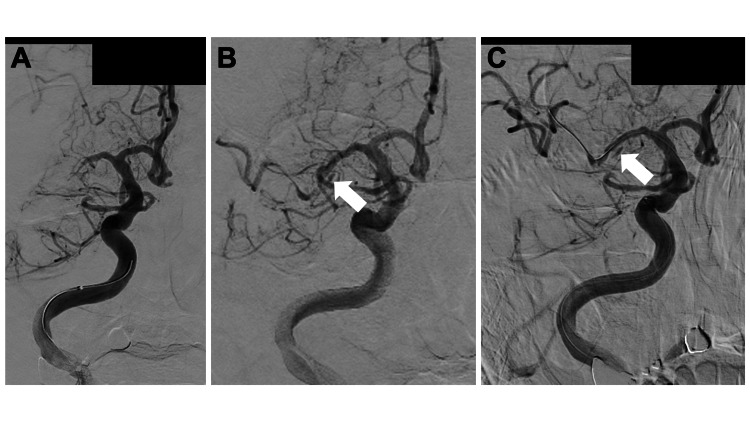
Angiographical findings (A) Initial angiogram showing right M1 occlusion without evidence of dissection. The first pass was performed using a combined technique, and the right M1 with stenosis was recanalized. (B) At this point, a double lumen already existed (white arrow), but second-pass and percutaneous transluminal angioplasty were performed without being noticed. (C) A double lumen is detected in the right M1 on the final angiogram (white arrow).

The patient underwent MT on the same day. An 8Fr EMBOGUARD™​ Balloon Guide Catheter (Cerenovus, Johnson & Johnson MedTech, Irvine, California, United States) was guided to the right internal carotid artery (ICA), which was occluded at M1 of the right MCA (Figure 2A). Subsequent imaging showed no evidence of dissection in the right ICA, and a combination of a Trevo Trak ® 21 Microcatheter (Stryker Corporation, Kalamazoo, Michigan, United States) microcatheter and an ASAHI CHIKAI® black 14 soft tip (Asahi Intecc Co Ltd, Aichi, Japan) microguidewire was advanced beyond the occlusion site. The thrombus was partially retrieved using a combination of RED 68 KIT Reperfusion Catheter (Penumbra, Alameda, California, United States) and Solitaire™ X Revascularization Device - 4mm x 40mm (Medtronic plc, Galway, Ireland) devices.

Although partial recanalization was achieved, severe stenosis was noted in the mid-to-distal region of the right M1, leading to re-occlusion after 5 minutes. Upon examination of the images post-procedure, a double lumen suggestive of an MCAD was noted; nevertheless, the surgeon did not recognize this finding and attempted a second pass, assuming acute occlusion owing to atherothrombotic brain infarction (ATBI) (Figure 2B, white arrow). A subsequent pass was sought but failed to alleviate the stenosis. The patient then underwent percutaneous transluminal angioplasty (PTA) using a Gateway PTA Balloon Catheter 2.0×9 mm (Stryker Corporation) to address the residual stenosis, again under the assumption of ATBI. The final digital subtraction angiography (DSA) image confirmed the presence of a double lumen in the right M1 segment (Figure 2C, white arrow). No subarachnoid hemorrhage (SAH) was observed on Xper CT (Koninklijke Philips N.V., Amsterdam, Netherlands). 

Simultaneous three-dimensional CT angiography and MRA performed on postoperative days (PODs) 3 and 26, respectively, did not reveal any discernible alteration in M1 occlusion or obvious aneurysmal dilatation. Follow-up MRI exhibited a sizable infarction in the right cerebral hemisphere without evidence of SAH. The patient was transferred to a rehabilitation facility with a modified Rankin scale (mRS) grade of 4 on day 44.

## Discussion

No apparent ICA dissection was observed in this case, indicating isolated MCAD localized to the MCA. During MT, no resistance was encountered while traversing the obstruction with a microguidewire or microcatheter, allowing the tip of the wire to be maneuvered freely. If dissection had developed during the procedure instead of at onset, the patient would have complained of a severe headache; however, in this case, the patient consistently reported a headache since arrival. The surgeon was unaware of any intraoperative arterial dissection, and the patient experienced a headache from the initial examination, leading to the diagnosis of isolated MCAD. The authors disregarded the possibility of a dissecting lesion as the headache at onset was deemed inconsequential, and the site of occlusion was atypical for an arterial dissection. Upon identifying dissection during MT, we discontinued the treatment with inadequate recanalization owing to the concern that the emergence of SAH might exacerbate the condition.

Isolated MCAD is a rare clinical condition with only scattered individual reports (Table 1) that can manifest as ischemia or rupture of a dissected aneurysm, leading to SAH or intracerebral hemorrhage with a high mortality rate [3]. If MCAD presents as an MCA occlusion, its frequency is 2.4% of all MCA occlusions [3]. Nounaka et al. reviewed 80 cases of MCAD, including 74 cases that met the inclusion criteria and six cases from their own experience [1]. Of these cases, 36 (45%) were ischemic, and there were no significant differences in age, sex, or medical history between patients with hemorrhage and those with ischemia. Lin et al. also investigated the differences between isolated MCAD and MCAD originating from ICA dissection (ICAD-MCAD) [7]. They observed that ischemia was more prevalent than hemorrhage in both conditions and that both were responsible for stroke at a young age, with an average age of 22 years. The mortality rates were high and similar, with most cases being idiopathic. However, they noted that congenital vessel wall defects are more common in ICAD-MCAD, whereas trauma is more frequently associated with isolated MCAD.

Triggers of isolated MCAD

Common vascular risk factors such as hypertension, smoking, diabetes mellitus, hyperlipidemia, and oral contraceptives are considered risk factors for intracranial artery dissection [8]. Additionally, many other conditions are associated with MCAD, including migraine, fibromuscular dysplasia, cystic intimal necrosis, intimal fibroelastic irregularities, homocystinuria, periarteritis nodosa, syphilitic arteriopathy, moyamoya disease, atherosclerosis, Guillain-Barré syndrome, Marfan’s syndrome, and Ehlers-Danlos syndrome [2]. Similar risk factors may be present in isolated MCAD; however, one characteristic of MCAD is its anatomical location in the sphenoidal wing. M1 is the most commonly dissected part of the MCAD, and friction between M1 and the sphenoidal wing may cause dissection owing to its proximity to the sphenoidal wing [2].

Symptoms of isolated MCAD

According to a prior review, headache is a common symptom of MCAD [2], and nearly 20% of patients do not exhibit neurological symptoms [8]. Asaithambi et al. reported 61 cases of isolated MCAD in their review, with 33 cases involving ischemia, of which 44% presented with headaches [2]. However, the study also included trauma and hemorrhage and did not specify the exclusive frequency of headaches in cases of ischemia. The frequency of headaches in acute cerebral infarction ranges from 8% to 34% as per Seifert et al. [5], and 8% to 18% in cerebral embolism [9]. Headaches are more prevalent in lesions affecting the posterior circulation than in those affecting the anterior circulation. In essence, the possibility of MCAD increases when acute MCA occlusion is associated with headache. However, Zhang et al. reported a case of isolated MCAD without headache, emphasizing that MCAD cannot be ruled out in the absence of a headache [8]. Moreover, in cases of acute ischemia, interviewing patients about headaches may be challenging owing to impaired consciousness or aphasia.

Diagnosis of isolated MCAD

DSA is the most used imaging modality for diagnosing intracranial artery dissection, followed by CT, MRI, pathological examination, and transcranial Doppler [10]. DSA is considered the gold standard [3]. Typical findings include string-like signs, irregular stenosis, pseudoaneurysms, and complete occlusion. In a review by Nounaka et al., DSA was performed in 76 patients (95%), including three patients diagnosed with MCAD based on MRI changes over time [1]. However, definitive diagnoses with typical DSA findings have been obtained in <10% of cases, indicating the difficulty of diagnosis [3]. In most cases, the flap is not depicted and is recognized as a stenosis [2]. An SVS-like finding with heterogeneous signal in M1 has been documented on MRI as a T2* shadow sign [11]. The finding of an MRI T2*-weighted image in this instance is considered to correspond to this T2* shadow sign. Additionally, there are scattered reports indicating that MRA source images are effective for detecting flaps and double lumens [12]. Recently, high-resolution MRI (HRMRI) has been employed for the diagnosis of MCAD. Kwak et al. performed 3T MRI-based HRMRI in three cases of MCAD, verifying lumen patency and flap; in one instance, a hematoma was visualized in the false lumen [13]. Despite the potential of HRMRI as a noninvasive diagnostic method, it is constrained by several technical limitations, such as overestimation of stenosis and artifacts [8]. 

Treatment of isolated MCAD

The optimal therapeutic intervention for Isolated MCAD remains unclear [8]. Treatment options during the chronic phase encompass antiplatelet therapy, anticoagulation therapy, thrombolytic therapy, surgery, endovascular therapy, and conservative therapy. Nevertheless, there is no consensus on the hyperacute treatment of patients with complete MCA occlusion. The safety of intravenous recombinant tissue-type plasminogen activator (rt-PA) in intracranial artery dissection remains undetermined [8,14,15]. Doijiri et al. documented that 0.6 mg/kg of intravenous rt-PA was administered to patients with MCAD with ischemic onset, and no apparent complications were observed [16]. Although extensive studies on MT are lacking, Park et al. reported seven cases of MT for M1 occlusion resulting from acute MCAD [3]. Following the initial fatality owing to SAH from guidewire perforation, they performed ADAPT (A Direct Aspiration, First Pass Technique) in subsequent patients, resulting in favorable outcomes, emphasizing the utility of ADAPT, particularly in cases with a conspicuous intimal flap, where microguidewire passage might be ill-advised; in one case, stenting was also performed.

In the present case, stenting was not performed; however, stenting could have been considered if the true lumen had been securely maintained by the guidewire. In the report by Nanatsue et al., an ENTERPRISE™ 2 Vascular Reconstruction Device (Cerenovus) was successfully placed to address acute M1 obstruction caused by isolated MCAD [17]. Two cases of iatrogenic MCAD arising during MT were managed with a coronary stent and Enterprise VRD [18], indicating the effectiveness of stenting in treating isolated MCAD. However, intracranial stenting is not reimbursed by insurance in Japan, necessitating prior consultation with an in-house ethics committee. Furthermore, there have been documented instances of rapid symptom and imaging improvement with antiplatelet medication, even in the hyperacute phase [8]. Therefore, careful evaluation of the need for surgical intervention is crucial.

A summary of cases with MCAD reported in the literature has been given in Table 1. 

**Table 1:** Summary of MCAD cases reported in the literature GR: good recovery; MCAD: middle cerebral artery dissection; MT: mechanical thrombectomy; rt-PA: recombinant tissue-type plasminogen activator; SAH: subarachnoid hemorrhage

Author(s)	Year	Case	Treatment	Outcome
Nounaka et al.[1]	2023	6 cases of MCAD, 1 with hemorrhage and 4 with aneurysms	No MT cases. Conservative treatment or standby surgery (bypass in 2 cases and aneurysm removal in 1)	All achieved GR
Asaithambi et al. [2]	2014	61 cases of MCAD, including 33 cases of ischemic stroke	No MT cases. Most ischemic cases were treated with antithrombotic drugs, while surgery was performed in 4 cases.	Not Available
Park et al. [3]	2020	7 cases of MCAD	MT in 6 patients, MT & stent in 1 patient	1 patient died of SAH, while 5 achieved GR.
Zhang et al. [8]	2018	1 case of MCAD	Rapid improvement after oral antiplatelet therapy.	GR
Shimohata et al. [12]	2014	1 case of MCAD	Antithrombotic therapy	GR
Doijiri et al. [16]	2012	1 case of MCAD	Intravenous rt-PA therapy	GR
Nanatsue et al. [17]	2024	2 cases of MCAD	Enterprise VRD	GR
Nakahara et al.[18]	2021	2 cases of iatrogenic MCAD	Stenting with coronary stent and Enterprise VRD	GR

## Conclusions

We presented a case of acute M1 occlusion caused by isolated MCAD. The onset of a headache may be a hint for suspicion, although distinguishing this rare condition is challenging. There is no definitive consensus on treatment. Given the potential fatality of SAH owing to dissection progression, a cautious approach to treatment is warranted. Additional case accumulation is imperative to determining the optimal management strategy.
